# Bibliometric Reports for Institutions: Best Practices in a Responsible Metrics Scenario

**DOI:** 10.3389/frma.2021.696470

**Published:** 2021-06-30

**Authors:** Alvaro Cabezas-Clavijo, Daniel Torres-Salinas

**Affiliations:** ^1^ Faculty of Business And Communication Studies, Universidad Internacional de La Rioja (UNIR), Logrono, Spain; ^2^ Department of Information And Communication Sciences, Universidad de Granada, Granada, Spain

**Keywords:** bibliometrics, reports, best practices, Responsible metrics, Responsible research and innovation

## Abstract

Carrying out bibliometric reports is one of the common tasks performed by librarians and practitioners within the framework of their professional duties. The emergence of novel data sources, the need to measure new research activities and the growing demand for fairer and more equitable evaluation within the framework of the Responsible Metrics movement has led to calls for a review of the traditional approaches to these types of reports. The main goal of this study is to outline a series of recommendations for bibliometricians, consultants and research support librarians when drafting bibliometric reports in their institutions. These best practices can significantly enhance the quality and utility of bibliometric reports, posing their practitioners as key players in the science management process.

## Introduction

In recent years, the evaluation of the performance of research institutions has become an increasingly complex task for universities, research centers and funding and evaluating bodies around the world. The emergence of novel data sources, the measurement of new research activities beyond the mere publication of scientific results and the increasing need for fairer, more equitable and responsible assessment procedures have led to a new scenario characterized by multidimensional evaluations that consider aspects such as knowledge transfer, the diversity of research outputs that an institution can generate and other ethical, integrity and equity issues. These aspects call for a rethinking of the traditional bibliometric reports, i.e., those that mainly analyze results in scientific journals and use citation indexes such as Web of Science or Scopus, which are produced or commissioned by research institutions (for example, Universidad de Granada, 2014; Barcelona Institute of Science and Technology, 2019).

### Bibliometric Units

The growing demand for proven bibliometric information and the increasing complexity of research measurement processes has generated the appearance in R and D centers and universities of departments specializing in the evaluation of scientific activity, the so-called ‘bibliometrics units’ or ‘science evaluation units’, among other names. These units may be configured in very different ways, with very different roles and tasks depending on the needs of each institution. The functions performed by these units include (Torres-Salinas and Jiménez-Contreras, 2012): a) management of research information sources b) generation of analysis, prospective and surveillance reports and c) training, advice and expert consultation. Table 1 highlights some of the bibliometrics units that have been created in recent years in Spain, following in the footsteps of the pioneering Bibliometrics Department of the University of Vienna launched in 2009.

**TABLE 1:** Examples of Bibliometrics Units in Spanish universities. Source: Own Elaboration.

University	Name of the department/unit	Year
University of Granada	Unidad de Evaluación de la Actividad Cientifica (Scientific Activity Evaluation Unit)	2011
University of Las Palmas	Unidad de Bibliometria (Bibliometrics Unit)	2013
University of Navarre	Unidad de Bibliometria (Bibliometrics Unit)	2014
University of Seville	Unidad de Bibliometria (Bibliometrics Unit)	2018
University of Cadiz	Unidad de Bibliometria (Bibliometrics Unit)	Not Av.
University of the Basque Country	Unidad de Bibliometría–Observatorio de la Produccion Científica (Bibliometrics Unit - Scientific Production Observatory)	Not Av.

One of the most important tasks of practitioners (research support librarians, research analytics librarians, liaison librarians, research performance analysts, bibliometrics officers, consultants, bibliometricians, etc.), whether in the framework of higher education institutions or working in consulting firms, is the preparation of bibliometric reports. These quantitative reports tend to have a descriptive purpose, that is, they aim to reflect the state of the research at a given moment, for example in a university, or an evaluative purpose, for example if the report is used to support the assessment of a certain funding call or area of the institution.

The Department for Bibliometrics and Publication Strategies of the University of Vienna is a good example of a unit which prepares both descriptive and evaluative reports, using its own methodology (Gumpenberger et al., 2012; Gorraiz et al., 2020). Similar activities are carried out by different institutions across the world, such as the University of New South Wales in Australia (Drummond and Wartho, 2009), the Technical University of Munich in Germany (Leiß 2017) and Universidad San Ignacio de Loyola in Peru (Pacheco-Mendoza et al., 2020). A special case is the Center for Science and Technology Studies (CWTS) at Leiden University in the Netherlands, which has become a key provider of bibliometric assessment reports for a wide range of institutions at an international level (Petersohn and Heinze, 2018) through its company CWTS BV. It is also necessary to highlight the role that numerous consulting firms have played in the preparation of bibliometric and evaluation reports, such as Science-Metrix, Technopolis, Evidence LTD, Digital Science Consultancy, EC3metrics and the Institute for Scientific Information (ISI), re-established in 2018 as the analytics expertise service of Clarivate Analytics.

Whether consulting firms or bibliometrics units, the preparation of these kinds of documents requires a number of specific skills (Iribarren-Maestro, 2018): knowledge of the different publication and citation guidelines in the different scientific areas; application of knowledge regarding statistics, scientific policies, legislation and other matters to the analysis and interpretation of results; recognition of the characteristics of the publications of scientific journals and publication models; identification of the characteristics of editorial quality products, and insights into the different university rankings depending on the nature of the reports requested. According to the competency model for those supporting bibliometrics (Cox et al., 2019), tasks associated with the design and execution of bibliometric reports–such as evaluation of departmental/research center performance, or assessment of institutional performance–are considered as ‘specialist tasks’ by the professional community, the highest level of competency for bibliometric work (Cox et al., 2019).

Depending on the needs of each institution, different types of reports may be generated (Torres-Salinas and Jiménez-Contreras, 2012; Gorraiz et al., 2020):

• Bibliometric reports at an institutional level: the results of these reports may be included in annual reports, with the main goal being to provide a precise overview of the state of the research at a particular point in time.
• Case studies: bibliometric reports focusing on a certain aspect of the research which is of strategic interest to the institution. For example, they may focus on a specific topic (gender, collaboration, open access) or a specific area (engineering, arts, fine arts, biomedicine).
• Decision-making and supporting reports: these provide useful information for scientific policymakers, such as reports for specific funding calls, faculty evaluations, recruitment or appointment procedures.
• Informative bibliometric reports: intended for the dissemination of key research findings by the University Communications Office to the media and the general public.

### The Responsible Metrics Scenario

Bibliometric professionals should also be aware of the Responsible Metrics movement and associated international manifestos and recommendations calling for the responsible use of bibliometric indicators. This matter has been gaining repercussion in recent years and more and more institutions are integrating some of these fundamental principles in their evaluation policies. The two main documents defining the responsible use of evaluation indicators are the San Francisco Declaration on Research Assessment (DORA, 2012) sponsored by the American Society for Cell Biology and the Leiden Manifesto for Research Metrics (Hicks et al., 2015) issued by a number of renowned bibliometric experts. These documents call for a more balanced and fairer approach to the use of metrics in research evaluation, especially with regard to individuals (recruitment, staff promotion, scholarships, calls for mobility, grants, etc.). Bibliometric practitioners cannot ignore this perspective when designing and executing these types of studies, even though it could be argued it is “time-consuming, expensive and requires a significant increase in bibliometric expertise” (Coombs and Peters, 2017). It is especially relevant for practitioners affiliated with institutions which are signatories of these manifestos. According to the 2019 Responsible Metrics State of the Art Survey (Robinson-García and Gadd, 2019), 23% of the respondents belonged to institutions that have signed DORA.

### Purpose of This Study

**FIGURE 1:**
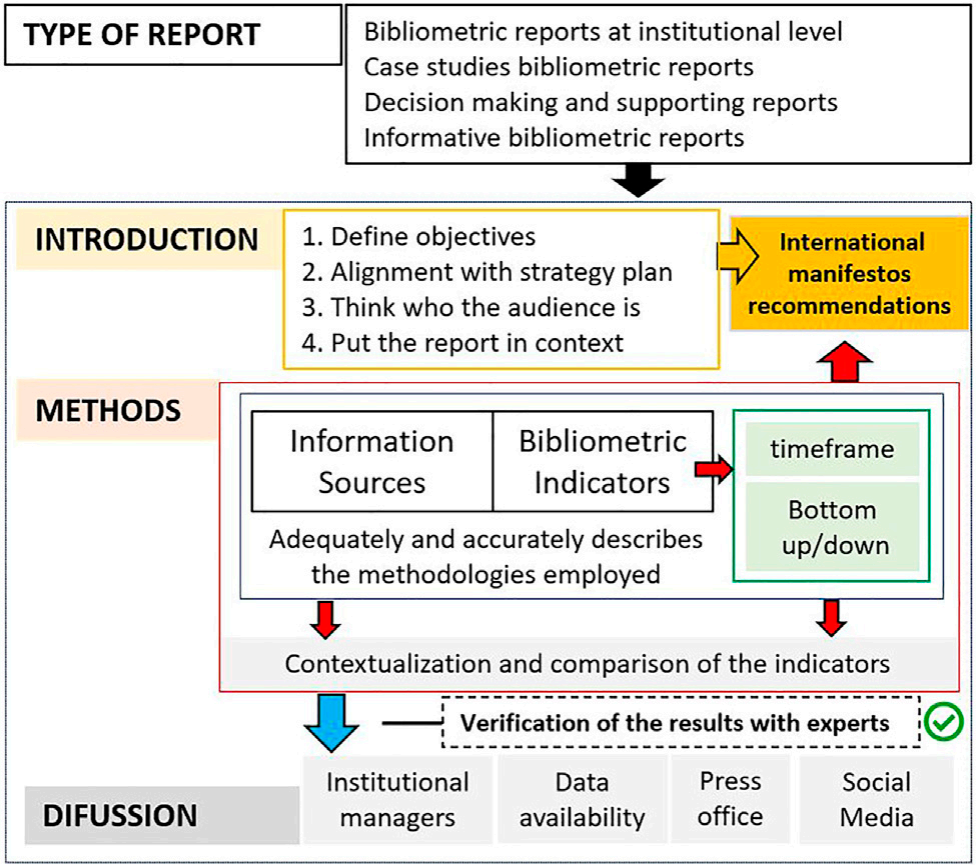
“Flowchart of the main processes and decisions for bibliometric reporting at an institutional level in a Responsible Metrics scenario”.

In light of this professional scenario, the main purpose of this study is to establish a series of best practices and recommendations for bibliometricians, consultants and research support librarians when drafting bibliometric reports for their institution. These guidelines are intended mainly for generation of bibliometric reports at an institutional level and case studies, although some of them are also applicable to evaluative reports to support decision-making. These recommendations are based on an extensive number of reports generated by different universities, consulting firms and bibliometric experts and are also guided by the framework offered by the Responsible Metrics principles. Figure 1 provides a concise summary of the reporting process and the decisions we will need to make in order to prepare bibliometric reports. In *Best Practices for Bibliometric Reports* we explain each of these actions and processes in greater detail.

## Best Practices for Bibliometric Reports

A series of recommendations have been set out below as a guideline to follow when preparing a bibliometric report. They include different international recommendations and should be considered as a compendium of best practices with a special emphasis on bibliometric reports for R and D institutions. These ten recommendations may be divided into three different blocks. The first block includes preliminary aspects that introduce the report to the reader and is concerned with adequate definition of the objectives and correct introduction of the socioeconomic context of our institution. The second block compiles four recommendations relating to all the methodological aspects of the report. We will have to make multiple decisions, starting with the databases and indicators we are going to use. This block also includes advice on the importance of adequately describing the methods and contextualization/comparison of the results. Finally, the third block presents four best practices that are based on Responsible Metrics principles and the growing demand for transparency and accountability in modern society.

### Preliminary Matters

#### Define the Objectives

Any report must indicate the objectives of the analysis carried out, contextualizing it within the framework of other similar studies carried out by the same institution. It should also be indicated whether the report is regular (biannual, annual) or if it is part of a series. The orientation of the report (descriptive or evaluative) and the purpose of the study must be adequately broken down. It is essential for it to be duly aligned with the objectives of the institution, with the purpose of the report being linked to the goals designed in the strategic plan of the organization.

For example, if one of the objectives of the institution is to expand its international presence, this purpose may be matched to indicators referring to international publications or collaborations. The need for the use of metrics should be adequately explained, since it should not be overlooked that in certain contexts the use of bibliometric indicators may be seen “as a challenge to academic freedom and to the university’s traditional role as a center in society of critical and independent thinking” (Cox et al., 2019). Likewise, the target audience of the study should be indicated (research managers, media, wider public, institution staff), along with the use that may be made of it and the context in which the information included in the report may be used.

#### Provide a Socioeconomic Context for the Institution

Offer a context for the results presented. It is a good idea to devote a brief introductory chapter to the socioeconomic aspects of your organization to facilitate an understanding of the bibliometric indicators used. For example, information could be included on GDP, labor structure, employment rates, production sectors, R and D investment, university staff, students, etc. This context may explain or at least qualify and generate a better understanding of the results obtained. This contextual information is especially important for readers unfamiliar with the institution or who do not belong to its sphere of influence. For example, in the case of reports on university alliances, international research networks or multicenter research, a brief description of the social and economic environment of each institution can provide valuable information about the achievements reached by their components, since the starting points and goals of each node within the network may be very different.

### Methodological Aspects

#### Select and Describe the Used Indicators

One of the key aspects of a report is to determine which indicators are best suited to achieve the objectives. Define a set of indicators that measure different dimensions; reports that assess just one dimension of scientific activity, such as publications in top-ranked journals, without considering other variables (scientific impact, collaboration, training capacity, research funds, etc.) should be avoided. There are hundreds of indicators that allow us to offer a multidimensional view of the research. However, only use indicators which are validated by the scientific community through publication in peer reviewed outputs, and which are broadly used by bibliometric experts. . Use also metrics which are easy to interpret, as non-experts have difficulty understanding complex indicators. Avoid inventing your own indicators, especially composite metrics that mix several indicators in a single measure. Likewise, avoid conscious attempts to manipulate the results, for example choosing metrics that may clearly favor your institution or certain areas or researchers within it.

Always include in the institutional report a precise definition of any of the indicators you are using, describing particularly detailed calculations and/or formula and their advantages and shortcomings. Table 2 offers an example of how to describe the indicators. You can also draw inspiration from handbooks such as “The Evaluation of research by Scientometric Indicators” (Vinkler, 2010), “Applied Evaluative Infometrics” (Moed, 2017) or “Handbook of Bibliometric Indicators: Quantitative Tools for Studying and Evaluating Research” (Todeschini and Baccini, 2017) to help you choose the right indicator. Karolinska Institutet offers a good example of best practices for the description and use of bibliometric indicators at an institutional level. It would be desirable for all institutions to have documents like the “Bibliometric Handbook for Karolinska Institutet” (Rehn and Kronman, 2008) and “Bibliometric Indicators—Definitions and Usage at Karolinska Institutet” (Karolinska Institutet, 2014).

**TABLE 2:** Example of how indicators can be defined and described in a bibliometric report. Source: Own Elaboration based on Karolinska Institutet (2014).

	
Designation	Hirsch index
Abbreviation	H-Index
Definition	The h-index is the number of publications (h) attributed to the unit analyzed during the time span analyzed that have at least h citations.
Calculation and/or Formula	Find the unit’s published articles in a citation index and sort them in descending order by number of citations. Count articles from the top of the list downwards and when the number of an article rises above the citation count for that same article, the number of the preceding article is to be counted as the h-index.
Data Requirements	Requires data from a comprehensive citation database (Web of Science, Scopus or Google Scholar)
Advantages	➔ Very easy to calculate in different databases
	➔ Included in different research profiles (Google Scholar, Scopus ID, … )
	➔ Accepted and very well known by the scientific community
Disadvantages	➔ h-index gives positive bias to senior researchers with older articles
	➔ The indicator is not field-normalized, which makes it unsuitable for. ➔ comparisons between researchers in different research fields
Use and application	We use the h-Index to generate author rankings and detect the researchers with the greatest impact in different areas.
Reference	Hirsch, J. E. (2005). An index to quantify an individual’s scientific research output. Proceedings of the National Academy of Sciences of the United States of America, 102(46), 16569–16572.

Fortunately, nowadays most of the indicators we need can be found and calculated in the most popular bibliometric databases. Commercial suppliers (Clarivate Analytics, Elsevier, etc.) propose a large number of indicators in SciVal^1^ and InCites^2^ handbooks. In both cases, definitions, calculations and formulas are presented. The metrics offered on these platforms highlight the huge number of bibliometric indicators available. InCites has a total of 64 indicators classified into six sections (Productivity, Impact, Collaboration, Reputation, Open Access and Author Position). On the other hand, SciVal offers 29 bibliometric indicators classified into seven groups (Collaboration, Published, Viewed, Cited, Economic Impact, Societal Impact, and Awards). In the case of SciVal, mention should also be made of the Snowball Metrics Initiative (Colledge, 2017), which develops a set of standard methodologies to calculate research metrics in a consistent way regardless of the data sources.

Bibliometricians can also take advantage of new indicators such as altmetrics and social media metrics offered by a number of platforms, as they can provide valuable information to study new forms of interaction between the general public, scholars and academic stakeholders (Zahedi and Costas, 2018) and measure the broader impact of research. Bornmann (2014) identifies four benefits of altmetrics compared to traditional metrics: broadness, diversity, speed, and openness. Nevertheless, serious concerns have arisen regarding the meaning of these metrics and a number of limitations may also be identified concerning the data quality, such as bias, measurement standards, normalization and, replication (Bornmann, 2014).

#### Use the Appropiate Sources, Databases and Tools

Use a diverse range of databases, avoiding the use of single sources that show significant results only for a limited number of disciplines. Local or national bibliometric products should be used to complement areas that are not well covered by international databases, as occurs in the arts and social sciences. According to Jappe (2020), only one out of every four bibliometric assessment studies uses national sources. Current Research Information Systems (CRIS), as well as institutional administrative databases and other non-bibliometric sources, can offer a more precise picture of research in the institution and are critical to offer accurate and significant results. Nevertheless, using CRIS and internal databases (in relation to grants or human resources, for example) may require intense work with the institution’s administrators and a time-consuming curation process.

**FIGURE 2:**
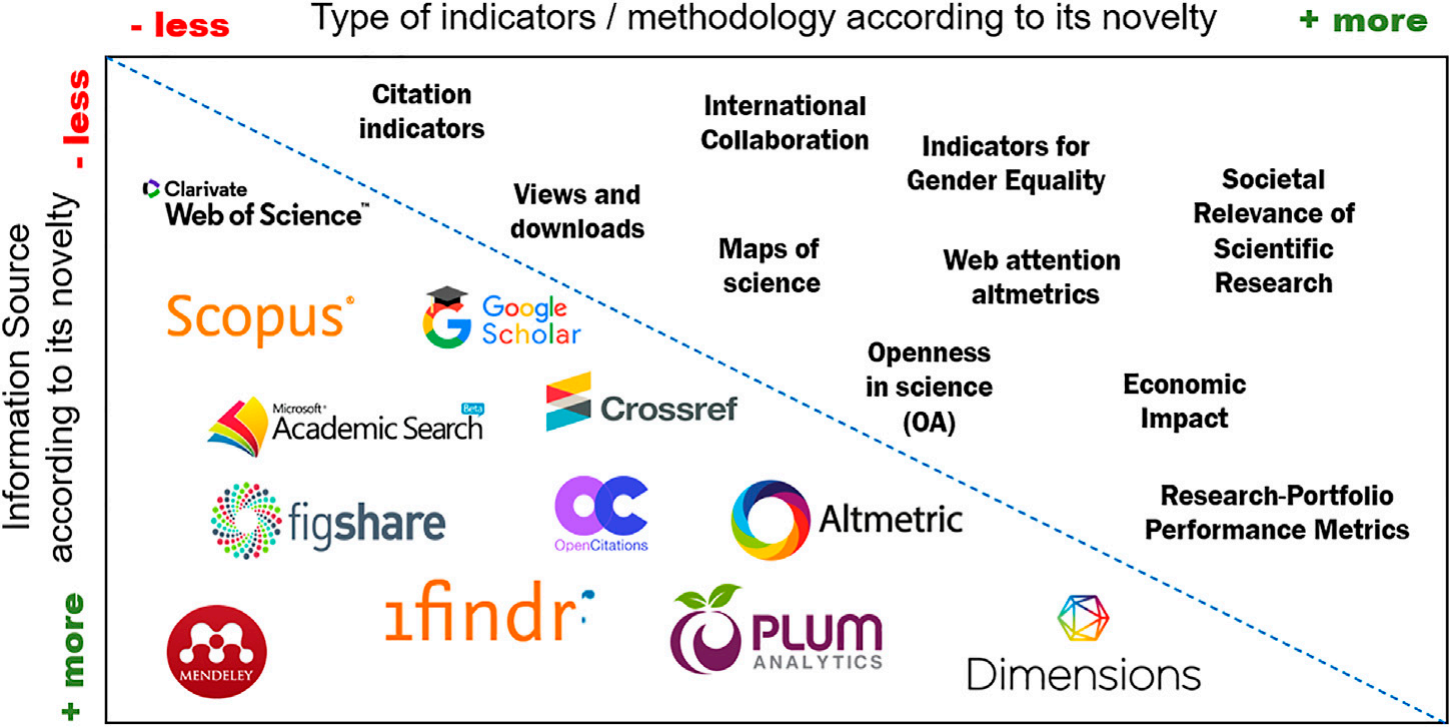
“Main information sources and indicators for bibliometric reports”.

News databases and altmetric sources (e.g., PlumX^3^ and Altmetric.com^4^) can provide relevant information on the outreach and communication activities of the institution and its social/societal impact, while university rankings (e.g. Leiden Ranking^5^, ARWU^6^ and Webometrics^7^) can provide information on the institution’s research impact and web visibility. The report work team should also be aware of the possibility of automatically collecting data from various sources *via* API. Figure 2 offers an overview of some of the information sources currently available and the indicators they allow us to calculate.

Another central issue to be determined by practitioners is the software used for data gathering and presentation of results. There are a number of bibliometric suites on the market developed by renowned companies such as SciVal^8^ (Elsevier), InCites^9^ (Clarivate) and Dimensions Analytics^10^ (Digital Science) that allow generation of results in various formats. There are also several free products that can also be helpful when preparing the full report or completing a specific section of it, such as Publish or Perish^11^, Bibliometrix^12^, Vosviewer^13^, and Scimat^14^, so these should be thoroughly evaluated. Moral-Muñoz, et al. (2020) provides a valuable review of the various tools available for conducting bibliometric and scientometric analyses.

#### Control the Methods

Clearly define the methodological aspects: chronological framework, approach, units of analysis, data collection methods, databases used, coverage, etc. The reports published by CWTS (2017) and the Nordic Institute for Studies in Innovation, Research and Education (NIFU, 2019) clearly address these issues. Point out the limitations of the study so that the results may be properly contextualized. Remember that it should be possible to reproduce and replicate all the aspects of the study. For example, regarding the unit of analysis, the aggregation level used should be stated. Three levels may be distinguished: a) micro-level, when the report focuses on individual researchers or research groups, b) meso-level, when it refers to departments or institutions, and c) macro-level, when the assessment is related to a region or country.

A further consideration is the way the indicators are compiled, i.e., bottom-up or top-down. Under the bottom-up approach, analysis begins with the data collection of the individual researchers of the institution (micro level) before moving up to higher aggregation levels by grouping the documents. This technique requires great precision in the compilation as well as verification by the researchers evaluated, and is the recommended procedure in reports aimed at decision-making by research managers. This approach also allows retrieval of documents that researchers have produced outside their current work centers (Costas, 2008). On the other hand, under the top-down approach the data is collected at an institutional level and the analysis may then be lowered to other more disaggregated levels. Data collection under this approach is much faster (since a search by affiliation can be done in selected data sources), although it lacks the accuracy and precision of the former approach, making it more appropriate for descriptive studies.

Use relatively long timeframes to observe the evolution of the indicators over time. A minimum period of five years of analysis is recommended. The use of short timeframes (two to three years) could overestimate some indicators which may be affected by a specific event or by specific legislation or regulations, thereby not duly reflecting the evolution and dynamics of a particular aspect of research within the institution. A useful technique to improve the stability of indicators that avoids changes in trends caused by a specific event is the use of overlapping periods (for example, 2017–2019; 2018–2020; 2019–2021). Likewise, caution must be exercised with the data of the most recent year, since they may be affected by updating procedures in the data sources, as well as by insufficient volume of information (e.g., citation window). Finally, we recommended maintaining stability over time in the methodologies used. In the case of annual reports, the same set of basic indicators should be used and avoid changing the data providers in order to facilitate comparability of annual trends.

#### Compare and Contextualize the Results

Always compare the results obtained with other institutions and contextualize them by region, country or thematic area in order to determine and understand the performance of your center. The use of comparisons and contextualization is of key importance to take full advantage of bibliometric information. Comparisons should be made with institutions with similar profiles, i.e., analogous size, objectives and disciplines. For example, a historical university with a general profile should not be compared with a technical university or a recently established center focusing on biomedical sciences. Use international benchmarks to contextualize the performance of the university or center such as Essential Science Indicators, or statistics reported by organizations such as the Organization for Economic Cooperation and Development (OECD), the United States National Science Foundation (NSF) or Eurostat at a European level. General and disciplinary baselines can be used to assist with in-depth interpretation of the information. Table 3 shows a real example of benchmarking for the University of Granada with three indicators, one absolute and two relative indicators.

**TABLE 3:** Example of contextualization and comparison of bibliometric indicators at an institutional level: University of Granada. Source: Own Elaboration.

1.A. Example of contextualization of the University of Granada with three baselines	Web of Science Documents	Category Normalized Citation Impact	% Documents in Q1 Journals
University of Granada	21,312	1.26	53.11%
Global Baseline	15,834,230	0.96	47.33%
EU-27 Baseline	4,031,472	1.1	50.20%
Spain—Baseline	548,508	1.2	55.46%

1.B. Example of comparison of the University of Granada with three universities	Web of Science Documents	Category Normalized Citation Impact	% Documents in Q1 Journals
University of Barcelona	45,919	1.68	62.70%
University of Granada	21,312	1.26	53.11%
University of Seville	18,890	1.05	53.40%
Complutense University of Madrid	26,902	1.12	52.29%

### Responsible Metrics Issues

#### Obtain Validation

Early drafts should be revised by a scientific committee of experts working in your institution, which can provide useful insights to improve the quality of the report and detect possible errors and inconsistencies. You can also ask for the support of policymakers or relevant researchers from different disciplines who can explain and qualify specific results involving unique publication and citation habits, or anomalous data, which may be determined by aspects relating to the sources used, legislative changes or socioeconomic conditions. When dealing with sensitive topics or especially relevant issues, an expert committee can be set up to guide and validate the data, methods, and procedures.

#### Pay Attention to Diversity

Be aware of the diversity of research areas present in the institution; avoid solely applying indicators intended for experimental or biomedical sciences. Consider research in local languages as well as activities that contribute to improve the socioeconomic environment in the area around the University or center analyzed. Avoid solely paper-focused reports. Bear in mind the Hong Kong Principles for Assessing Researchers (Moher et al., 2020) and try to introduce indicators aimed at valuing a broader range of research and scholarship, such as replication, innovation, translation, synthesis, and meta-research, peer review, mentoring, outreach, and knowledge exchange, among others.

#### Apply Ethical, Integrity and Equality Principles

Apply ethical, integrity and equality principles in accordance with the numerous international recommendations in this regard. Consider the latest developments in Responsible Research and Innovation and try to incorporate some of these new indicators in your analysis. For example, the SUPER MoRRI (Scientific Understanding and Provision of an Enhanced and Robust Monitoring system for Responsible Research and Innovation) Project^15^ identifies up to 36 indicators in six different areas: gender equality, literacy and science education, public engagement, ethics, open access and governance. Finally, any conflicts of interest that may arise should be disclosed.

#### Make the Report Public and Open Your Data

**FIGURE 3:**
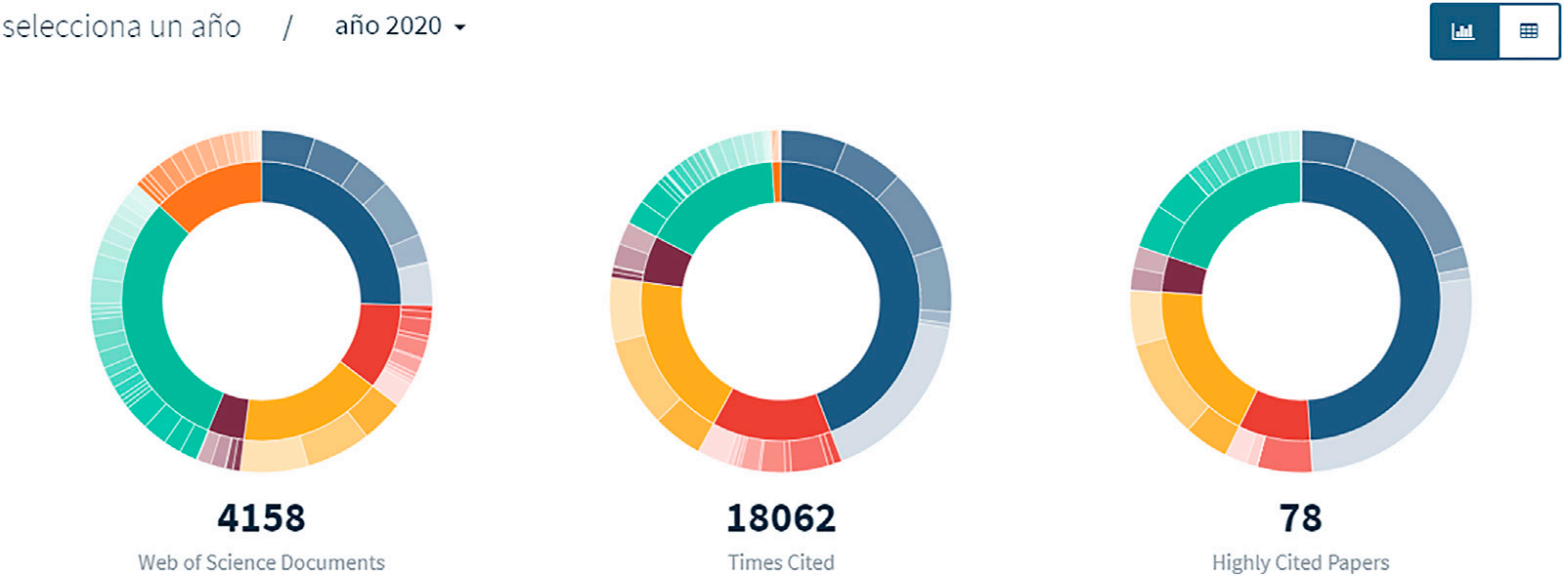
“Example of data visualization–LiveMetrics project”.

Make the results of the report available to the public, unless there is a confidentiality agreement to restrict the publication. Present the data in an attractive way through interactive reports, infographics or dedicated websites. For example, the LiveMetrics project^16^ of the University of Granada presents bibliometric indicators and R and D statistics for the University in a dynamic and up-to-date way (Figure 3). Also take advantage of general and academic social media and the University Communications Office to maximize the reach of your report. Make the raw data of the reports open and accessible to facilitate the replicability of the study and its reuse by other researchers. Upload your data to the Open Data platform of your institution or use an external repository.

## Final Remarks

The preparation of more responsible bibliometric reports within the framework of scientific policies that seek to be increasingly fair and equitable and more closely connected with the challenges of modern society constitutes a major challenge for librarians and evaluation specialists. This study has presented a series of recommendations for a new generation of bibliometric studies that definitively abandon dependence on single sources and the exclusive measurement of scientific articles, in favor of a broader vision that adequately evaluates the different forms of research carried out by universities and R and D centers.

We are aware that very few reports will be able to take into account all the variables suggested in this study, nonetheless the possibility exists for these types of analyses to move forward in the direction set by new trends in the responsible metrics scenario. The more professionals assume and implement these best practices, the greater the influence they will have in the science management process, offering relevant answers to the challenges posed by research activity today.

## Data Availability

The original contributions presented in the study are included in the article, further inquiries can be directed to the corresponding author.

## Footnotes

1
https://service.elsevier.com/app/answers/detail/a_id/13936/supporthub/scival/

2
http://help.prod-incites.com/inCites2Live/8980-TRS/version/default/part/AttachmentData/data/InCites-Indicators-Handbook-6%2019.pdf

3
https://plumanalytics.com/learn/about-metrics/

4
https://www.altmetric.com/

5
https://www.leidenranking.com/

6
http://www.shanghairanking.com/

7
https://www.webometrics.info/

8
https://www.scival.com/

9
https://incites.clarivate.com/

10
https://www.dimensions.ai/products/dimensions-analytics/

11
https://harzing.com/resources/publish-or-perish

12
https://www.bibliometrix.org/

13
https://www.vosviewer.com/

14
https://sci2s.ugr.es/scimat/

15
https://www.super-morri.eu/super-morri/index.php

16
https://livemetrics.ugr.es/
